# Supplementary material for: Differential Impact of Tumor Endothelial Angiopoietin-2 and Podoplanin in Lymphatic Endothelial Cells on HCC Outcomes with Tyrosine Kinase Inhibitor Treatment According to Sex
Source: Biomedicines. 2024 Jun 26;12(7):1424. doi: 10.3390/biomedicines12071424 (PMC11273995; doi:10.3390/biomedicines12071424)
Supplement: Supplementary file 1 [file biomedicines-12-01424-s001.zip › biomedicines-3045051-supplementary.pdf]

Table S1. Univariate Cox regression analysis for extra-hepatic spread.

	Univariate analysis	
**Variables**	**HR (95% CI)**	**p-value**
Endothelial podoplanin*	0.000 (0.000 – 0.159)	**0.011**
Time on TKI**	0.928 (0.865 – 0.996)	**0.040**
Age at diagnosis***	0.917 (0.875 – 0.961)	**0.000**
Sex	0.028 (0.000 – 3.734)	0.152
Tumoral Ang2	0.008 (0.000 – 4.217)	0.132
Endothelial Ang2****	0.000 (0.000 – 0.266)	**0.025**
Tumoral CLEC-2	0.096 (0.000 – 47.976)	0.460
HCC grade	1.365 (0.740 – 2.519)	0.319
Bilirubin	1.080 (0.494 – 2.359)	0.848
INR	1.833 (0.146 – 23.010)	0.639
Albumin	0.410 (0.092 – 1.819)	0.241
Creatinine	0.052 (0.003 – 0.962)	0.057
Platelet	1.000 (1.000 – 1.000)	0.054
AFP	1.000 (1.000 – 1.000)	0.965
Not-viral	1.242 (0.700 – 2.203)	0.458
Varici	0.698 (0.280 – 1.737)	0.439
PVT	1.565 (0.492 – 4.979)	0.448
*,**,***,**** collinear		
